# Antioxidant Potential and Antibacterial Efficiency of Caffeic Acid-Functionalized ZnO Nanoparticles

**DOI:** 10.3390/nano7060148

**Published:** 2017-06-16

**Authors:** Kyong-Hoon Choi, Ki Chang Nam, Sang-Yoon Lee, Guangsup Cho, Jin-Seung Jung, Ho-Joong Kim, Bong Joo Park

**Affiliations:** 1 Department of Electrical & Biological Physics, Kwangwoon University, 20 Kwangwoongil, Nowon-gu, Seoul 01897, Korea; solidchem@hanmail.net (K.-H.C.); gscho@kw.ac.kr (G.C.); 2 Department of Medical Engineering, Dongguk University College of Medicine, Gyeonggi-do 10326, Korea; kichang.nam@gmail.com; 3 Department of Chemistry, Gangneung-Wonju National University, Gangneung 25457, Korea; nanochemistry@naver.com; 4 Department of Chemistry, Chosun University, Gwangju 61452, Korea

**Keywords:** multifunctional nanoparticle, antioxidant activity, antibacterial activity, caffeic acid, ZnO

## Abstract

We report a novel zinc oxide (ZnO) nanoparticle with antioxidant properties, prepared by immobilizing the antioxidant 3-(3,4-dihydroxyphenyl)-2-propenoic acid (caffeic acid, CA) on the surfaces of micro-dielectric barrier discharge (DBD) plasma-treated ZnO nanoparticles. The microstructure and physical properties of ZnO@CA nanoparticles were characterized by field emission scanning electron microscopy (FESEM), transmission electron microscopy (TEM), infrared spectroscopy, and steady state spectroscopic methods. The antioxidant activity of ZnO@CA nanoparticles was evaluated using an ABTS (3-ethyl-benzothiazoline-6-sulfonic acid) radical cation decolorization assay. ZnO@CA nanoparticles exhibited robust antioxidant activity. Moreover, ZnO@CA nanoparticles showed strong antibacterial activity against Gram-positive bacteria (*Staphylococcus aureus*) including resistant bacteria such as methicillin-resistant *S. aureus* and against Gram-negative bacteria (*Escherichia coli*). Although Gram-negative bacteria appeared to be more resistant to ZnO@CA nanoparticles than Gram-positive bacteria, the antibacterial activity of ZnO@CA nanoparticles was dependent on particle concentration. The antioxidant and antibacterial activity of ZnO@CA may be useful for various biomedical and nanoindustrial applications.

## 1. Introduction

Antioxidants inhibit the oxidation process in biological systems and the environment by acting as free radical scavengers, reactive oxygen scavengers, or reducing agents [1,2]. Recently, a number of studies have investigated the antioxidant activities of synthetic as well as natural antioxidants [3,4,5]. Caffeic acid (CA) has been increasingly studied in pharmacological research owing to its antioxidant, cardiac, immunomodulatory, and anti-inflammatory activities [6,7,8]. CA is a natural phenolic acid widely found in plants and at high levels in some herbs, spices, and sunflower seeds. It is also one of the major natural phenols in argan oil.

In the pharmaceutical, biological, and food industries, natural antioxidants have been introduced into substrate materials to prevent or reduce oxidation in situations where free antioxidants cannot be used, such as under ambient O_2_, volatilization, and thermal instability [9,10,11]. Nanoparticles are now widely used throughout the pharmaceutical, catalyst, electronics, nano-patterning, and tissue engineering industries [12,13,14,15]. Nanoparticles often possess unique nanoscale size-dependent physical and chemical properties that can be controlled in a manner that is not possible in the bulk state. Among the various nanoparticles, ZnO nanoparticles have been extensively investigated over the past decade in the fields of materials science, biotechnology, and medical engineering for their potential applications as catalysts and chemical absorbents [16,17]. A previous in vitro study of ZnO nanoparticles showed that particle size, particle morphology, surface modifications, and reactivity in aqueous solutions determined their biocompatibility [18,19]. In particular, ZnO nanoparticles exhibit high antimicrobial activity even at low concentrations. They offer many advantages as an antibacterial agent because of their good stability at high temperatures and pressures and long shelf life when compared to organic antibacterial agents. By employing various strategies to modify and tailor ZnO nanoparticle surfaces, multifunctional nanoparticles with improved aqueous dispersion and biocompatibility have been developed that can be used for targeted drug delivery and bioimaging [20,21,22]. However, although considerable progress has been made in the preparation of multifunctional ZnO nanoparticles for biological applications, ZnO nanoparticles with antioxidant properties have not been reported.

In the present study, we describe a simple surface modification process that functionalizes 20 nm ZnO nanoparticles with the antioxidant CA. The antioxidant activity of the resulting ZnO@CA was evaluated using an ABTS (3-ethyl-benzothiazoline-6-sulfonic acid) radical cation decolorization assay and compared to that of free CA. The prepared ZnO@CA was tested for its antimicrobial activity against *Escherichia coli*, *Staphylococcus aureus*, and methicillin-resistant *S. aureus* (MRSA). The physical and structural properties of ZnO@CA nanoparticles were also investigated. To the best of our knowledge, this is the first demonstration of antioxidant and antibacterial activities by a functionalized ZnO nanoparticle. This novel multifunctional nanoparticle represents a promising material for therapeutic applications in biomedical engineering.

## 2. Results and Discussion

**Figure 1:**
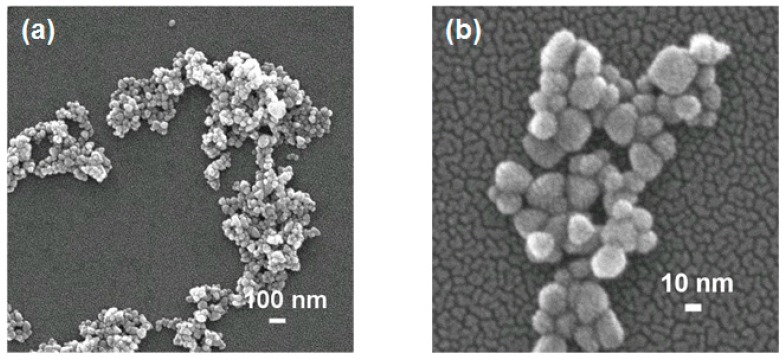
(**a**) Low and (**b**) high magnification FESEM images of ZnO@CA nanoparticles. The ZnO@CA nanoparticles have a spherical shape with a narrow size distribution. The mean size of the ZnO@CA nanoparticles is ~20 nm.

The morphology, structure, and size of ZnO@CA nanoparticles were investigated using FESEM, high resolution transmission electron microscopy (HRTEM), and X-ray diffraction (XRD). Figure 1 shows FESEM images of ZnO@CA nanoparticles synthesized at a molar ratio of 1:2 (Zn nitrate:KOH). FESEM images of ZnO@CA nanoparticles revealed a spherical shape with a narrow size distribution. They were assembled in aggregates, as shown in Figure 1a. The high-magnification SEM image shows very small ZnO@CA crystallites with various spherical shapes. The average size of the ZnO@CA nanoparticles was about 20 nm, which is consistent with the size of the nanoparticles determined by XRD analysis of Sherrer’s formula.

**Figure 2:**
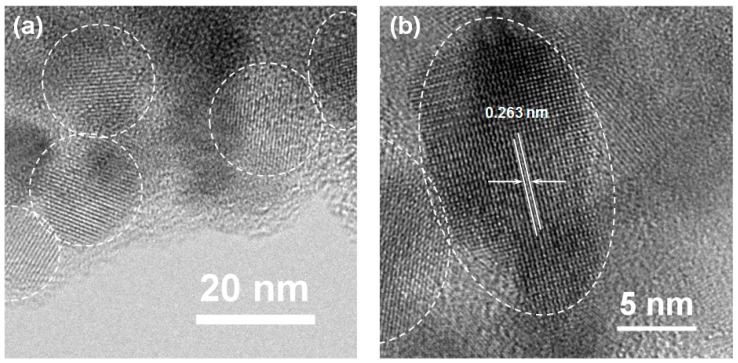
(**a**) Low magnification and (**b**) high magnification TEM micrographs of ZnO@CA nanoparticles. The interlayer distance of the lattice fringe is estimated to be ≈2.63 Å, which is comparable to those of the (002) planes in the typical wurtzite structure of ZnO.

The microstructure of the ZnO@CA nanoparticles was characterized using HRTEM. As illustrated in Figure 2a, many ZnO@CA nanoparticles with a single crystalline nature were confirmed in the agglomerated particles. The mean size of a single nanoparticle was approximately 20 nm (Figure 2a). In Figure 2b, the ZnO@CA nanoparticles are single crystalline with an appropriate distance of 2.63 Å between two neighboring planes. This crystalline property is consistent with the neighboring crystal planes (002) of the wurtzite structure of ZnO.

**Figure 3:**
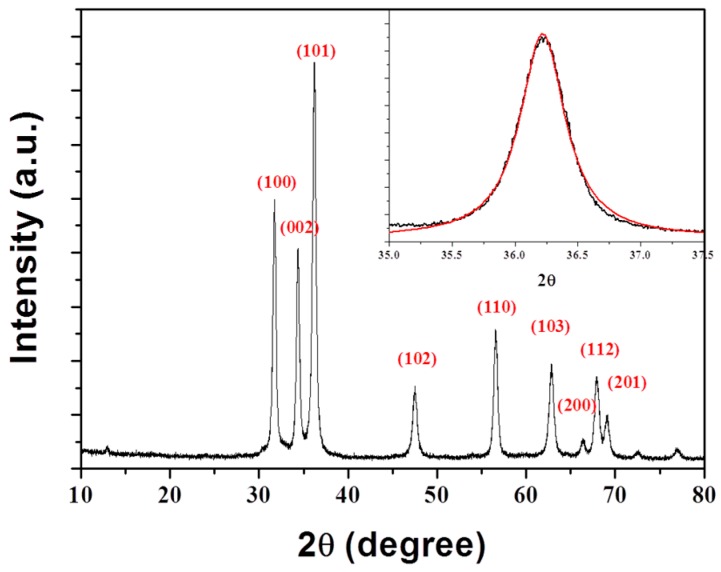
XRD spectrum of ZnO@CA nanoparticles. All diffraction peaks can be indexed to the wurtzite type lattice of ZnO, which matches well with the standard XRD pattern (JCPDS No. 36-1451).

Figure 3 shows a typical XRD pattern of ZnO@CA nanoparticles in the range of 10–80° at a scanning step of 0.01. A number of Bragg reflections with values of 31.7°, 34.3°, 36.2°, 47.5°, 56.5°, 62.8°, 66.3°, 67.9°, and 69.1° were observed corresponding to the (100), (002), (101), (102), (110), (103), (200), (112), and (201) planes, respectively [23]. All diffraction peaks in the XRD pattern can be indexed to the wurtzite ZnO structure (hexagonal phase, space group *P*63*mc*, and JCPDS No. 36-1451) [24]. The large amplitudes and small widths of the diffraction peaks indicate a high degree of crystallinity in ZnO@CA nanoparticles, as does the absence of other peaks in the XRD pattern, which might indicate the potential presence of impurities. To estimate the average crystallite size (D) of ZnO@CA nanoparticles, diffraction peak profiles were fit with a convolution of Lorentzian functions (inset of Figure 3), and the extent of line broadening was estimated using the Scherrer equation [25]

(1) $$D\;=\;\frac{0.9\lambda }{\beta \;\times \;cos\theta }$$

where D is the crystallite size (nm), *λ* is the wavelength of incident X-ray (0.154 nm), *β* is the full width at half maximum, and *θ* is the Bragg’s diffraction angle. The mean crystallite size of ZnO@CA nanoparticles was 19.3 nm, which was calculated using the most intense peak (101) in the XRD pattern.

**Figure 4:**
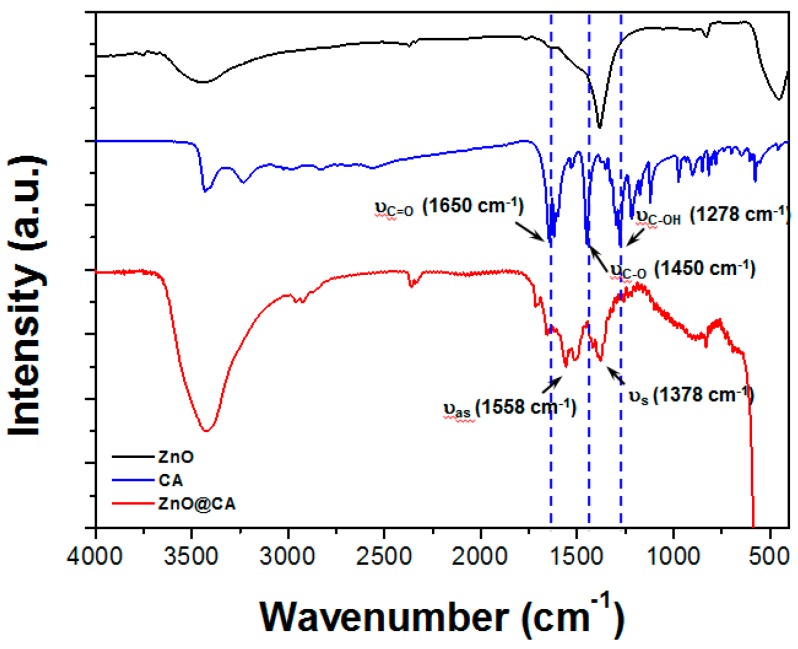
FT-IR spectra of free ZnO, CA, and ZnO@CA nanoparticles. The carboxylrate group of CA is bounded to the surface of the ZnO nanoparticle symmetrically through its two oxygen atoms.

To confirm that a covalent bond had formed between the carboxyl group of CA and the Zn ions on the surfaces of the ZnO nanoparticles, Fourier transform infrared (FT-IR) spectroscopy analyses of free ZnO, CA, and ZnO@CA nanoparticles were performed (Figure 4). First, the FT-IR spectrum of ZnO could not confirm any vibrational peaks of the CA molecules. Second, the IR spectrum of pure CA showed major absorption peaks at 1650, 1450, and 1278 cm^−1^, which corresponded to the stretching modes of the free carboxyl double bond (υ_C=O_), the carbon–oxygen single bond (υ_C-O_), and the O-H deformation (υ_C-OH_), respectively (top panel of Figure 4) [26]. Absorption peaks at these positions are consistent with the IR spectrum of protonated carboxyl groups (COOH). In contrast, the IR spectrum of ZnO@CA nanoparticles showed new peaks at 1558 and 1378 cm^−1^, which correspond to the asymmetric (υ_as_ = 1558 cm^−1^) and symmetric (υ_s_ = 1378 cm^−1^) stretching modes of the carboxylate group (bottom panel of Figure 4). These results indicate that the carboxyl group of CA is covalently bound to the Zn ions on the surfaces of ZnO nanoparticles.

ZnO@CA nanoparticles and free CA were evaluated for their ability to scavenge ABTS radicals. ZnO@CA nanoparticles were estimated to have on average four CA molecules per particle and exhibited favorable dispersion and stability in water. The abilities of ZnO@CA nanoparticles and free CA to scavenge ABTS radicals are shown in Table 1. Free CA scavenged ABTS radicals in a concentration-dependent manner, exhibiting its highest activity (93.25%) at the highest concentration tested (100 µM). The *o*-dihydroxybenzene moiety of CA exhibited high antioxidant activity because it is converted to stable *o*-quinone derivatives by reaction with ABTS radicals. At concentrations between 20 and 100 µM, ZnO@CA nanoparticles robustly scavenged ABTS radicals, with activities ranging from 44.99% to 73.68%, respectively. The reduced antioxidant activity of ZnO@CA nanoparticles compared to that of free CA may be attributed to steric repulsive forces between nanometer-sized ZnO and ABTS radicals. From these results, it was confirmed that the CA molecules were responsible for the antioxidant activity of the ZnO@CA nanoparticles. CA, which is a plant secondary metabolite, is well known as a natural antibiotic [27]. In addition, CA molecules are known to be biocompatible with a high affinity to cells. Therefore, the ZnO@CA nanoparticles will generate a significant synergy for the antimicrobial activity. In future, this functional coating technology could be an alternative for preventing microbism in clinical applications.

For a quantitative antibacterial test of ZnO@CA nanoparticles, we used a static culture method after mixing the bacterial solution and the nanoparticles due to the properties, which tend to sink to the bottom depending on their weight. With this method, we assessed the antibacterial activity of the nanoparticles without any target molecules on the surface of them.

**Figure 5:**
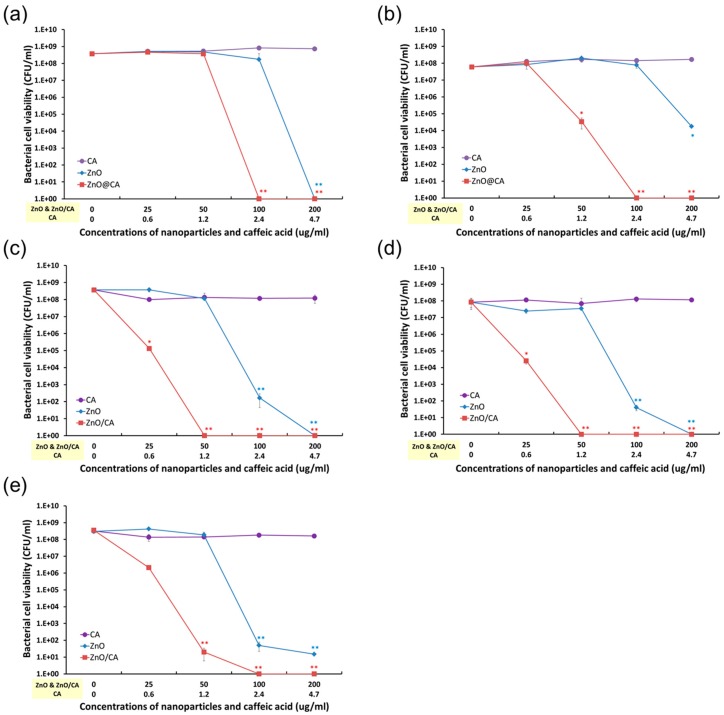
Antibacterial activities of ZnO@CA nanoparticles against Gram-negative and Gram-positive bacteria, including clinical isolates of methicillin-resistant *Staphylococcus aureus* (MRSA) strains. Antibacterial activities of the ZnO@CA nanoparticles against (**a**) *Escherichia coli*; (**b**) *Staphylococcus aureus*; (**c**) MRSA-1; (**d**) MRSA-2 (clinically isolated strain); and (**e**) MRSA-3 (clinically isolated strain). The killing efficiencies of the ZnO@CA nanoparticles against five bacterial strains, including clinical isolates of MRSA strains, are shown. The strains were incubated with the various concentrations of ZnO@CA nanoparticles and CA for 24 h. Data are expressed as means ± standard deviation (*n* = 6), as determined by the Student’s *t*-test. *p* < 0.05 was considered to indicate statistical significance (* *p* < 0.05, ** *p* < 0.005 vs. control).

**Figure 6:**
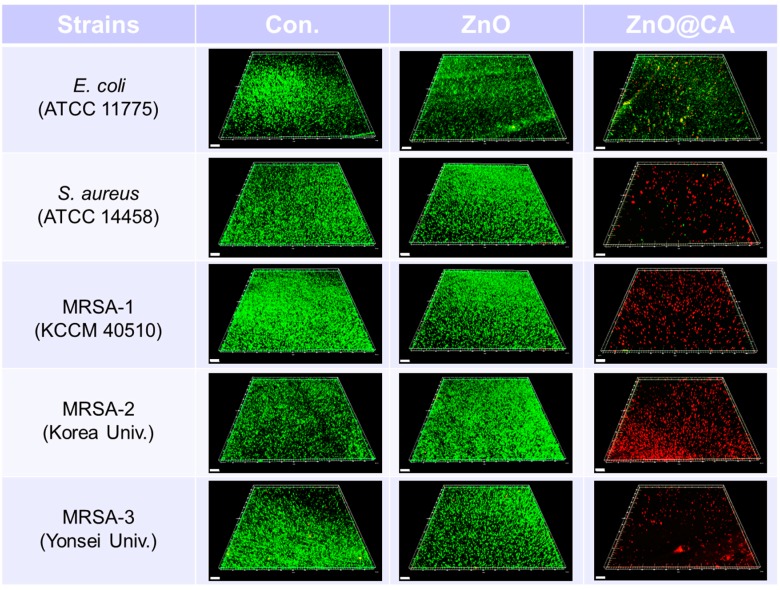
Live and dead cell images of bacteria (*E. coli*, *S. aureus*, MRSA-1, MRSA-2, and MRSA-3) using a confocal fluorescence microscope after incubation with ZnO and ZnO@CA nanoparticles. The bacterial cells of the five strains on the cover glass were incubated at 35 °C with ZnO and ZnO@CA nanoparticles for 24 h. The images show live and dead bacterial cells stained with SYTO-9 (green) and propidium iodide (PI, red) fluorescent dyes, respectively. All samples were tested in duplicate for each experiment, and each experiment was repeated three times (*n* = 6). There were no significant differences on live and dead cell imaging in each sample. Scale bars represent 50 μm.

The antibacterial activities of ZnO@CA nanoparticles were evaluated by measuring the total number of viable bacterial cells against five bacterial strains, including both Gram-negative and Gram-positive strains and three MRSA strains. As shown in Figure 5, ZnO@CA nanoparticles showed more effective antibacterial activity than CA or ZnO nanoparticles. CA had no antibacterial activity, whereas ZnO nanoparticles showed antibacterial activity against Gram-negative and Gram-positive bacteria at high concentrations (over 100 µg/mL). However, the ZnO@CA nanoparticles exhibited strong bacterial killing activities against both Gram-negative and Gram-positive bacterial cells. In particular, Gram-positive bacteria such as *S. aureus* and MRSA, including two clinical MRSA isolates (MRSA-2 and MRSA-3), were completely inhibited by the ZnO@CA nanoparticles, which showed perfect killing efficiency. The antibacterial efficiencies of the ZnO@CA nanoparticles were dependent on the cell type, as they inhibited the growth of MRSA (including clinically isolated strains) more efficiently than that of Gram-negative bacteria (*E. coli*). The ZnO@CA nanoparticles showed greater antibacterial activity against Gram-positive strains than against Gram-negative bacteria with high selectivity. The highly selective antibacterial activity against Gram-positive bacteria, particularly against MRSA, was also confirmed by confocal fluorescence microscopy images (Figure 6). The images show live and dead bacterial cells stained with SYTO-9 (green) and propidium iodide (PI, red) fluorescent dyes, respectively. *S. aureus* and clinical isolates of MRSA were completely killed by the ZnO@CA nanoparticles, and many dead MRSA cells were observed after only 2 h of incubation. However, some *E. coli* cells survived after incubation with ZnO@CA nanoparticles. The fluorescent images of live and dead cells show the selectivity of the ZnO@CA nanoparticles for the Gram-positive bacterial cells of *S. aureus* and MRSA strains.

Overall, the results described above suggest that the ZnO@CA nanoparticles not only exhibit potent antioxidant activity but also completely inhibit the growth of Gram-positive bacteria, particularly MRSA, with high selectivity, although, the ZnO@CA nanoparitcles need to confirm their biocompatibility in vitro and in vivo and the functionality also have to be tested on the pathogenic bacterium in vivo in the future.

## 3. Materials and Methods

### 3.1. Preparation of ZnO@CA Nanoparticles

ZnO nanoparticles with a diameter of 20 nm were prepared using previously described methods [28]. In a typical preparation, 0.03 M Zn(NO_3_)_2_·6H_2_O was dissolved in 110 mL ethanol and agitated ultrasonically for 15 min. After 15 min, 40 mL KOH ethanol solution was added under vigorous stirring (the molar ratio of Zn(NO_3_)_2_ to KOH was fixed at 1:2). The solution was maintained at room temperature (RT) for 1 h until the solution temperature dropped. After reflux reaction at 80 °C for 10 h and returning the solution temperature to RT, the resulting amber-colored precipitate was separated by centrifugation, washed with deionized water and absolute alcohol several times, and then dried in a vacuum oven at 60 °C for 6 h.

In order to conjugate CA molecules, the surface of ZnO nanoparticles were treated with micro-dielectric barrier discharge (DBD) plasma for 30 min under an electrical discharge power of approximately <3 W (0.7 kV, 5 mA, and phase angle of ~1 radian), as previously reported [29]. Antioxidant functionality of the ZnO nanoparticles was provided by a wet chemical process with CA as follows. ZnO nanoparticles prepared in ethanol (EtOH) (20 mg/mL) were mixed with a 2.08 × 10^−5^ M solution of CA/EtOH and agitated at RT for 24 h. After 24 h, the product was washed with EtOH several times. After the final wash, residual EtOH was further removed, and the product was dried at 60 °C for 12 h.

### 3.2. Physical Characterization of ZnO@CA Nanoparticles

The morphology and size of ZnO@CA nanoparticles were determined by field emission scanning electron microscopy (FESEM, SU–70, Hitachi, Tokyo, Japan) and transmission electron microscopy (TEM, JEM-2100F, JEOL, Tokyo, Japan). Crystallographic properties of ZnO@CA nanoparticles were investigated with an X-ray diffractometer (XRD, X’ Pert Pro MPD, PANalytical, Almelo, The Netherlands) operated at 40 kV and 150 mA in a 2*θ* range of 20–80°. IR spectra were obtained using a PerkinElmer Spectrum 100 FT-IR spectrometer.

### 3.3. Evaluation of Antioxidant Activity of ZnO@CA Nanoparticles

ABTS assays were performed using the following methods described by Arnao et al., but with modifications [30]. The ABTS radical cation (ABTS•+) was produced by mixing solutions of 7 mM ABTS and 2.4 mM potassium persulfate at equal ratios and incubating the solution at RT in the dark for 24 h. The solution was then diluted with methanol, and the absorbance was measured with a spectrophotometer until an absorbance of 0.7–1 units at a wavelength of 734 nm was achieved. Different concentrations of ZnO@CA nanoparticles were added to diluted ABTS•+ solutions for 30 min at 37 °C in the dark, after which the absorbance of the solution at a wavelength of 734 nm was measured using a spectrophotometer. The ability of ZnO nanoparticles to scavenge ABTS•+ radicals was calculated using the equation

% inhibition = [1 − (Absorbance of sample/Absorbance of control)] × 100(2)

### 3.4. Assessment of Antibacterial Activity of ZnO@CA Nanoparticles against Bacterial Cells

To confirm the antibacterial activity of the ZnO@CA nanoparticles, we used five strains of bacteria including three strains of MRSA, as follows. Two strains each of the Gram-negative and Gram-positive bacteria *E. coli* ATCC 11775 and *S. aureus* ATCC 14458 were purchased from the American Type Culture Collection (ATCC, Rockville, MD, USA). MRSA-1 (KCCM 40510) was obtained from the Korean Culture Center of Microorganisms (KCCM, Sedaemun-Gu, Seoul, Korea). Two clinically isolated MRSA strains were acquired: MRSA-2, isolated from patients at the Korea University Anam Hospital, and MRSA-3, isolated at the Yonsei Medical Center in Seoul, Korea, were both kindly donated by the respective hospitals [31,32].

*E. coli* (ATCC 11775) and *S. aureus* (ATCC 14458) were grown on plate count agar (PCA, Becton, Dickinson and Company, Sparks, MD, USA), and the three MRSA strains were grown on Brain Heart Infusion agar (BHIA, Becton, Dickinson and Company, Sparks, MD, USA) at 35 °C for 24 h. All bacterial strains were passaged twice at 48 h intervals before use.

For assessing the antibacterial activity of ZnO@CA nanoparticles, we used two methods: a quantitative method and a qualitative method. For the quantitative method, bacterial cells from each bacterial colony were suspended at ~10^6–7^ colony forming units (CFU)/mL in nutrient broth for *E. coli* and *S. aureus* and in BHI broth for MRSA strains. Bacterial cells in each broth solution were diluted 10-fold to ~10^5^–10^6^ CFU/mL and incubated with various concentrations of CA, ZnO, and ZnO@CA nanoparticles in 24-well plates at 35 °C for 24 h in static condition. After incubation with the samples, the bacterial cells in each well were inoculated onto BHI agar after serial 10-fold dilutions (10 to 10^7^) and incubated for 24 h. Bacterial cell viability was determined by counting the CFU, and the antibacterial activity of the ZnO@CA nanoparticles was determined by plotting the total number of viable bacterial cells as CFU/mL vs. the concentrations of the samples. Antibacterial activity of the ZnO@CA nanoparticles was defined as a >3 log decrease in CFU/mL.

The antibacterial activity of the ZnO@CA nanoparticles was confirmed with a confocal fluorescence microscope using live and dead bacterial cell images, after staining with two kinds of fluorescent dyes, green for live cells and red for dead cells. Each bacterial strain was suspended in normal saline solution. Bacterial cells at ~10^6^–10^7^ CFU/mL were inoculated onto sterilized cover glasses coated with poly-l-lysine in 24-well plates and incubated for 1 h to allow cells to attach to the cover glasses. Suspended bacterial cells were discarded after incubation, and the cover glasses in each well were gently rinsed three times with sterilized 0.9% saline solution to remove unattached bacterial cells. Bacterial cells on the cover glasses were incubated with ZnO and ZnO@CA nanoparticles for 24 h. After incubation, live and dead bacterial cells on the cover glass were stained with LIVE/DEAD BacLight Bacterial Viability Kits (Molecular Probes, Eugene, OR, USA) according to the manufacturer’s instructions. Live and dead bacterial cells were analyzed with a laser scanning confocal fluorescence microscope (FV-1200, Olympus, Tokyo, Japan) with 20× objective lenses and fluorescence optics (excitation at 485 nm for SYTO 9 and PI and emission at 530 nm for SYTO 9 and 630 nm for PI). Confocal fluorescence images of live and dead bacterial cells were analyzed using imaging software (Imaris, Bitplane, Concord, MA, USA).

### 3.5. Statistical Analysis

All samples were tested in duplicate for each experiment, and each experiment was repeated three times (*n* = 6). Quantitative data are expressed as the means ± standard deviation (SD). Statistical comparisons were performed with a Student’s *t*-test. A value of *p* < 0.05 was considered statistically significant.

## 4. Conclusions

In summary, we have for the first time, successfully fabricated ZnO nanoparticles with antioxidant and antibacterial activities. The ZnO nanoparticles were fabricated by covalently conjugating CA to ZnO nanoparticles using by a simple surface modification method. ZnO@CA nanoparticles with an average diameter of 20 nm were synthesized and characterized by FESEM, TEM, FT-IR, and XRD. Importantly, their antioxidant and antibacterial activities are robust and well suited for applications in biological technologies. In the future, nanoparticles of various sizes could be functionalized with widely used natural antioxidants and with specific targeting ligands to produce multifunctional nanomaterials for use in bioimaging and as antibacterial and anticancer therapeutic agents in the pharmaceutical industry.

## Figures and Tables

**Table 1:** ABTS radical scavenging activity.

Sample	Concentration (µM)	% Inhibition
CA:ZnO	20	44.99 ± 0.48
CA:ZnO	40	73.68 ± 2.51 ^a^
CA:ZnO	100	51.88 ± 3.56 ^a^
Caffeic acid	20	47.98 ± 0.72
Caffeic acid	40	78.62 ± 0.73 ^a^
Caffeic acid	100	93.25 ± 0.43 ^a,b^

Results are shown as the means ± SD of three independent experiments. Significant differences between groups were determined using a one-way ANOVA with post-hoc Tukey’s test, and *p* < 0.05 was considered significant. ^a^ Significant compared to activity at the lowest concentration of the same sample; ^b^ Significant difference between the two different samples at the same concentration.

## References

[1] Skerget M., Kotnik P., Hadolin M., Hras A.R., Simonic M., Knez Z. (2005). Phenols, proanthocyanidins, flavones and flavonols in some plant materials and their antioxidant activities. Food Chem..

[2] Gupta V.K., Sharma S.K. (2006). Plants as natural antioxidants. Nat. Prod. Radiance.

[3] Ito N., Hirose M., Fukushima S., Tsuda H., Shirai T., Tatematsu M. (1986). Studies on antioxidants: Their carcinogenic and modifying effects on chemical carcinogenesis. Food Chem. Toxicol..

[4] Mariod A.A., Ibrahim R.M., Ismail M., Ismail N. (2010). Antioxidant activities of phenolic rich fractions (PRFs) obtained from black mahlab (*Monechma ciliatum*) and white mahlab (*Prunus mahaleb*) seedcakes. Food Chem..

[5] Erkan N., Ayranci G., Ayranci E. (2008). Antioxidant activities of rosemary (*Rosmarinus officinalis* L.) extract, blackseed (*Nigella sativa* L.) essential oil, carnosic acid, rosmarinic acid and sesamol. Food Chem..

[6] Prasad N.R., Karthikeyan A., Karthikeyan S., Reddy B.V. (2011). Inhibitory effect of caffeic acid on cancer cell proliferation by oxidative mechanism in human HT-1080 fibrosarcoma cell line. Mol. Cell. Biochem..

[7] Hirose M., Takesada Y., Tanaka H., Tamano S., Kato T., Shirai T. (1998). Carcinogenicity of antioxidants BHA, caffeic acid, sesamol, 4-methoxyphenol and catechol at low doses, either alone or in combination, and modulation of their effects in a rat medium-term multi-organ carcinogenesis model. Carcinogenesis.

[8] Chen J.H., Ho C.T. (1997). Antioxidant activities of caffeic acid and its related hydroxycinnamic acid compounds. J. Agric. Food Chem..

[9] Scoponi M., Cimmino S., Kaci M. (2000). Photo-stabilisation mechanism under natural weathering and accelerated photo-oxidative conditions of LDPE films for agricultural applications. Polymer.

[10] Giannakopoulos E., Christoforidis K.C., Tsipis A., Jerzykiewicz M., Deligiannakis Y. (2005). Influence of Pb(II) on the radical properties of humic substances and model compounds. J. Phys. Chem. A.

[11] Deligiannakis Y., Sotiriou G.A., Pratsinis S.E. (2012). Antioxidant and antiradical SiO_2_ nanoparticles covalently functionalized with gallic acid. ACS Appl. Mater. Interfaces.

[12] Zhao F., Yao D., Guo R., Deng L., Dong A., Zhang J. (2015). Composites of polymer hydrogels and nanoparticulate systems for bomedical and pharmaceutical applications. Nanomaterials.

[13] Pardo-Yissar V., Katz E., Wasserman J., Willner I. (2003). Acetylcholine esterase-labeled CdS nanoparticles on electrodes: Photoelectrochemical sensing of the enzyme inhibitors. J. Am. Chem. Soc..

[14] Park S.J., Taton T.A., Mirkin C.A. (2002). Array-based electrical detection of DNA with nanoparticle probes. Science.

[15] Lin J., Raji A.R., Nan K., Peng Z., Yan Z., Samuel E.L., Natelson D., Tour J.M. (2014). Iron oxide nanoparticle and graphene nanoribbon composite as an anode material for high-performance Li-ion batteries. Adv. Func. Mater..

[16] Zhou Y., Fang X., Gong Y., Xiao A., Xie Y., Liu L., Cao Y. (2017). The interactions between ZnO nanoparticles (NPs) and α-linolenic acid (LNA) complexed to BSA did not influence the toxicity of ZnO NPs on HepG2 cells. Nanomaterials.

[17] Chen L., Xu J., Holmes J.D., Morris M.A. (2010). A facile route to ZnO nanoparticle superlattices: Synthesis, functionalization, and self-assembly. J. Phys. Chem. C.

[18] Li Z., Yang R., Yu M., Bai F., Li C., Wang Z.L. (2008). Cellular level biocompatibility and biosafety of ZnO nanowires. J. Phys. Chem. C.

[19] Zheng Y., Li R., Wang Y. (2009). In vitro and in vivo biocompatibility studies of ZnO nanoparticles. Int. J. Mod. Phys. B.

[20] Wu Y.L., Lim C.S., Fu S., Tok A.I., Lau H.M., Boey F.Y., Zeng X.T. (2007). Surface modifications of ZnO quantum dots for bio-imaging. Nanotechnology.

[21] Chakraborti S., Joshi P., Chakravarty D., Shanker V., Ansari Z.A., Singh S.P., Chakrabarti P. (2012). Interaction of polyethyleneimine-functionalized ZnO nanoparticles with bovine serum albumin. Langmuir.

[22] Xiong H.M. (2013). ZnO nanoparticles applied to bioimaging and drug delivery. Adv. Mater..

[23] Nagarajan S., Kuppusamy K.A. (2013). Extracellular synthesis of zinc oxide nanoparticle using seaweeds of gulf of Mannar, India. J. Nanobiotechnol..

[24] Zhang R., Kerr L.L. (2007). A simple method for systematically controlling ZnO crystal size and growth orientation. J. Solid State Chem..

[25] Choi K.H., Wang K.K., Shin E.P., Oh S.L., Jung J.S., Kim H.K., Kim Y.R. (2011). Water-soluble magnetic nanoparticles functionalized with photosensitizer for photocatalytic application. J Phys. Chem. C.

[26] Park B.J., Choi K.H., Nam K.C., Min J.E., Lee K.D., Uhm H.S., Choi E.H., Kim H.J., Jung J.S. (2015). Photodynamic anticancer activity of CoFe_2_O_4_ nanoparticles conjugated with hematoporphyrin. J. Nanosci. Nanotechnol..

[27] Maddox C.E., Laur L.M., Tian L. (2010). Antibacterial activity of phenolic compounds against the phytopathogen *Xylella fastidiosa*. Curr. Mocrobiol..

[28] Shao R., Sun L., Tang L., Chen Z. (2013). Preparation and characterization of magnetic core–shell ZnFe_2_O_4_@ZnO nanoparticles and their application for the photodegradation of methylene blue. Chem. Eng. J..

[29] Park B.J., Choi K.H., Nam K.C., Ali A., Min J.E., Son H., Uhm H.S., Kim H.J., Jung J.S., Choi E.H. (2015). Photodynamic anticancer activities of multifunctional cobalt ferrite nanoparticles in various cancer cells. J. Biomed. Nanotechnol..

[30] Arnao M.B., Cano A., Acosta M. (2001). The hydrophilic and lipophilic contribution to total antioxidant activity. Food Chem..

[31] Lee K.Y., Park B.J., Lee D.H., Lee I.S., Hyun S.O., Chung K.H., Park J.C. (2005). Sterilization of *Escherichia coli* and MRSA using microwave-induced argon plasma at atmospheric pressure. Surf. Coat. Technol..

[32] Choi K.H., Lee H.J., Park B., Wang K.K., Shin E., Park J.C., Kim Y., Oh M.K., Kim Y.R. (2012). Photosensitizer and vancomycin-conjugated novel multifunctional magnetic particles as photoinactivation agents for selective killing of pathogenic bacteria. Chem. Commun..

